# An evolutionary view of the *Fusarium* core genome

**DOI:** 10.1186/s12864-024-10200-w

**Published:** 2024-03-22

**Authors:** Daniel A. Gomez-Chavarria, Alvaro L. Rua-Giraldo, Juan F. Alzate

**Affiliations:** 1 Departamento de Microbiología y Parasitología, Facultad de Medicina, Centro Nacional de Secuenciación Genómica - CNSG, Sede de Investigación Universitaria (SIU), Universidad de Antioquia, Carrera 53 No. 61-30 Lab. 510, Medellín, Colombia; 2 Escuela de Microbiología, Universidad de Antioquia, Medellín, Colombia

**Keywords:** *Fusarium*, Phylogenomics, Comparative genomics, Evolution, Genome evolution

## Abstract

*Fusarium*, a member of the Ascomycota fungi, encompasses several pathogenic species significant to plants and animals. Some phytopathogenic species have received special attention due to their negative economic impact on the agricultural industry around the world. Traditionally, identification and taxonomic analysis of *Fusarium* have relied on morphological and phenotypic features, including the fungal host, leading to taxonomic conflicts that have been solved using molecular systematic technologies. In this work, we applied a phylogenomic approach that allowed us to resolve the evolutionary history of the species complexes of the genus and present evidence that supports the *F. ventricosum* species complex as the most basal lineage of the genus. Additionally, we present evidence that proposes modifications to the previous hypothesis of the evolutionary history of the *F. staphyleae*, *F. newnesense*, *F. nisikadoi*, *F. oxysporum*, and *F. fujikuroi* species complexes. Evolutionary analysis showed that the genome GC content tends to be lower in more modern lineages, in both, the whole-genome and core-genome coding DNA sequences. In contrast, genome size gain and losses are present during the evolution of the genus. Interestingly, core genome duplication events positively correlate with genome size. Evolutionary and genome conservation analysis supports the F3 hypothesis of *Fusarium* as a more compact and conserved group in terms of genome conservation. By contrast, outside of the F3 hypothesis, the most basal clades only share 8.8% of its genomic sequences with the F3 clade.

**Supplementary Information:**

The online version contains supplementary material available at 10.1186/s12864-024-10200-w.

## Background

Fungi can behave as saprophytes, endophytes, and pathogens, but only a few represent a risk to other living beings [1]. *Fusarium* is an Ascomycota that encompasses several relevant pathogenic species of plants and animals, including humans [2]. Some phytopathogenic species are notoriously responsible for economic losses valued at billions of dollars per year for the agricultural industry around the globe due to their potential to generate devastating disease outbreaks in almost any crop (cereals, vegetables, ornamental plants, fruits, flowers, etc.) [3, 4].

*Fusarium* species also stand out because of their ability to produce mycotoxins, which contaminate agricultural products, rendering them unsafe for human or animal consumption. Several of these mycotoxins have been associated with cancer and hormonal disorders in humans and farm animals [5]. Moreover, some *Fusarium* species can cause disease in humans, ranging from onychomycosis, skin infections, and keratitis in immunocompetent individuals to invasive or disseminated infections, mainly in neutropenic and immunosuppressed patients [6]. This genus's wall components and cell metabolites have been implicated in allergic processes in hypersensitive individuals [7]. It is estimated that *Fusarium* species can comprise more than 400 phylogenetically distinct species, most discovered in the last 25 years [8]. The Phylogenetic Species Concept (PSC) defines a species as the smallest diagnosable cluster of individual organisms within which there is a parental pattern of ancestry and descent, and which form a monophyletic lineage. In the context of fungi, the PSC is often referred to as Genealogical Concordance Phylogenetic Species Recognition (GCPSR). This approach involves analyzing multiple gene genealogies to identify concordant genealogical patterns that define distinct evolutionary monophyletic lineages corresponding to species [9].

In recent decades, the agricultural sector witnessed, with fear, the re-emergence of *Fusarium*, with the appearance *of Fusarium oxysporum f.sp. cubense* tropical race 4 (Foc TR4). This pathogen started to affect the Cavendish banana crops of this cultivar around the 1970s and now is present in all continents where banana is grown causing millions in losses. Cavendish cultivar was the solution to the appearance of the *F. o. fsp cubense* (Foc) race 1, the *Fusarium* species responsible for the well-known Panama disease, that was initially described in Australia in 1874 and destroyed all monoculture of banana cultivars ‘Gros Michel’ around the globe by the beginning of the twentieth century [10].

The destructive power of *Fusarium* is not restricted to crop devastation, some species produce secondary metabolites similar to mycotoxins, that can be toxic to humans and animals, including gibberellins and the mycotoxins fusarins, fumonisins, and trichothecenes [2, 5, 11, 12]. The fungus produces these toxins on stored agricultural products, or even directly on the growing plant [13]. During 1930 – 1940, in the Volga and Ural regions, fusariotoxins in winter cereals claimed the lives of tens of thousands of people [14].

*Fusarium* as a taxonomic group was first described in 1809 by Johann Heinrich Friedrich Link (Link, 1809). However, it went unnoticed until the publication "Die Fusariem" by Wollenweber and Reinking in 1935, where 65 species, 55 varieties, and 22 forms of *Fusarium* were described [15]. Historically, other alternative generic names have been proposed for *Fusarium* isolates based on the morphology of sexual stages like *Gibberella*, *Nectria*, and *Neocosmospora*. Nonetheless, only the *Fusarium* genus name should be used to avoid misunderstandings [2]. More than 400 phylogenetically distinct species in 23 monophyletic species complexes are included in the genus *Fusarium,* although not all have been formally designated [16].

Traditionally, *Fusarium* species classification relies heavily on morphological and phenotypical characteristics, which includes the affected host organism. The macro and microscopic structures analyzed are highly variable, and similarities between closely related species set the origin for several taxonomic inconsistencies observed until the first decade of this century. Genetic and biochemical features were added to resolve morphological disagreements with the scientific and technological developments at the end of the twentieth century [8, 9, 17–19]. Nevertheless, it was not until recent years, using modern phylogenetic methods, that evolutionary approaches clarified Fusaria's chaotic taxonomic assignment [3, 12, 14, 20, 21]. These first phylogenetic works were based on multilocus sequence analysis of conserved genes or partial genes like *cmdA*, *rpb1, rpb2*, *tef1*, and *tub2* [12, 20]. One of the noteworthy successes reached with molecular phylogenetics is recognizing *Fusarium* as a monophyletic group and setting the initial hypothesis of evolutionary relationships within the genus [12].

Even using molecular systematics, many published phylogenies have been conflictive or debated, especially those that have proposed major nomenclatural changes. Sometimes, it has been challenging to draw the limit between species and infraspecific lineages, and some results do not offer enough solidity to explain the evolutionary history of the genus, representing a problem for the academic community of *Fusarium* [3, 12].

With this work, we aimed to contribute to a deeper understanding of *Fusarium's* evolutionary relationships and various genomic features of *Fusarium* reference strains, including GC content, genome size, genome and core proteome conservation, and ancestral gene duplication events. Our results, derived from a phylogenomic approach utilizing 559 conserved proteins, provide evidence supporting the *F. ventricosum* species complex as the most basal lineage within the genus. Additionally, our study offers a novel perspective on the evolutionary history of the *F. sta*phyleae, *F. newnesense*, *F. nisikadoi*, *F. oxysporum*, and *F. fujikuroi* species complexes.

## Methods

### Reference genomes

The NCBI-Datasets website was consulted for *Fusarium* genomes on 06/06/22.

We filtered the results by the criterion "reference genomes RefSeq" and obtained 224 genomes [22]. In July of the same year, a new reference genome of the *ventricosum* species complex was added, *Fusarium robinianum* CBS430 (GCA_024115165), ultimately totalling 225 *Fusarium* reference genomes. The assemblies were subsequently processed with the BUSCO v3 program [23] to obtain additional genome quality metrics. Additionally, the reference genome that we originally accessed on June/22, downloaded as *F. ventricosum* NRRL 25729 (GCA_013623725), was removed from the database, and a new entry with the same genome was added as *F. robinianum* NRRL 25729 (GCA_013623725.1). We used this new RefSeq genome entry and the *ventricosum* species complex is represented by two *F. robinianum* genomes, strains NRRL 25729 and CBS430. The detailed list of the accession numbers of the genomes used in this study (*n* = 225), as well as the descriptive statistics of the assemblies can be found in Supplementary Table 1.

To filter out low-quality *Fusarium* genomes, we performed a boxplot graphics to identify outliers, allowing us to assess the dispersion range of genome metrics. The analyzed metrics included assembly length (assembled genome size), assembly N50, largest scaffold, scaffold count, BUSCO completeness, BUSCO single copy genes, BUSCO duplicated genes, BUSCO fragmented genes, and BUSCO missing genes. For the phylogenomic analysis, nine underperforming *Fusarium* assemblies were excluded (refer to Supplementary Methods for a detailed list). The exclusion criterium was BUSCO completeness index below 90%.

### Proteome annotation and filtering

Using the BUSCO [23] hypocreales_odb10 reference and the AUGUSTUS genome annotator (with *Fusarium graminearum* reference training set) [24, 25], we identified a set of ancestral conserved proteins of the *Fusarium* reference genomes. The same annotation strategy was applied to each genome. We used the default BLAST searches E-value cutoff of AUGUSTS; which is 1e-03. The annotated proteins in the genome of *Fusarium oxysporum* GCF_000271745, recognized by BUSCO as single-copy proteins, were compared using BLASTP [26] against a database of the ancestral conserved proteins of the BUSCO hypocreales_odb10 databank (*n* = 4,494). Proteins exceeding the filtering criteria (e-value = 0, %id = 80, and bit score ≥ 600) were retained as candidates for the phylogenomic analysis, totalling 996. These proteins underwent annotation using the EGGNOG-MAPPER web server [22]. Sequences with eggNOG orthologous group assignments that did not coincide with Hypocreales, family Nectriaceae, or those presenting a duplicated KEGG KO assignment code were excluded. Following this process, a set of 559 proteins was selected as markers for the phylogenomic analysis (refer to Supplementary Table 2).

### Phylogenomic analysis

To construct the 559-protein alignment super-matrix, we used a combination of BLASTP searches and individual MAFFT alignments [23]. In brief, the 559 filtered and curated proteins from the reference strain *Fusarium oxysporum* GCF_000271745 served as queries to identify the respective ortholog in each proteome using BLASTP. Subsequently, each set of orthologous proteins underwent individual alignment with the MAFFT aligner. The resulting 559 individual protein alignments were concatenated using the program catsequences (https://github.com/ChrisCreevey/catsequences). For the phylogenomic reconstruction, nine low-quality entries of *Fusarium* were excluded (see Supplementary Methods). As outgroups, we included six *Neonectria* reference species: *Neonectria hederae* (GCA003385265), *Neonectria punicea* (GCA003385315), *Neonectria coccinea* (GCA019137265), *Neonectria faginata* (GCA019137275), *Neonectria galligena* (GCA013759035), and *Neonectria ditissima* (GCA019137815).

One maximum likelihood phylogenomic tree was computed using IQ-TREE v2 [27], performing 5000 UFB pseudoreplicates [28] and using the different partitions models [29] for each protein with the options -m MFP + MERGE and -rcluster 15. The partition scheme used can be found in the supplementary material. The Log-likelihood of the consensus tree was -7,060,983.436. Furthermore, we computed gene concordance factors (gCF) and site concordance factors (sCF) using IQTREE v.2.2.0.8, employing the "–gcf" and "–scf" options to assess the level of genealogical agreement. gCF is defined as the proportion of gene trees that include a specific branch considered "decisive" for each branch of a species tree, while sCF is defined as the percentage of decisive alignment sites supporting that particular branch.

### Genome GC content analysis

To investigate GC content evolution, we employed both whole-genome GC content and core-genome coding DNA sequences (CDS). The calculation of core-genome CDS GC content involved utilizing single-copy CDSs predicted through BUSCO and Augustus. The number of core CDSs annotated for each genome fell within the range of 4076 to 4480. Consequently, the analysis of core genome GC evolution was performed with a minimum of 4076 CDS per genome. For this analysis, we omitted nine genomes that exhibited poor performance. The comprehensive list of excluded genomes is provided in the Supplementary Methods.

### Genome-to-genome comparisons and Average Amino Acid Identity (AAI) analysis

Genome alignment and comparative analysis were conducted using the DNADIFF program from the MUMMER v4 software [30]. All *Fusarium* genomes were aligned with each other, and the fraction of aligned bases and average nucleotide identity were extracted from the '.report' file. Subsequently, a non-redundant table was created and imported into R program for further analysis.

The Average Amino Acid Identity score was calculated using the EzAAI program [31]. To do this, single-copy proteomes annotated with BUSCO [23] were used as input for the comparisons. An all-vs-all comparison of single-copy proteomes was performed, and a non-redundant summarized table containing the AAI score values and proteome coverage ratio for all comparisons was constructed and utilized for the statistical analysis.

### Statistical analysis and graphics

All analyses and graphics, including boxplots, scatter plots and quantile analyses, were conducted using R and RStudio v.4.1.3 platforms, with the assistance of the ggplot2 library [32, 33]. Graphics, including boxplots and scatter plots, were generated using the ggplot2 package. Phylogenetic trees were visually edited using FigTree v1.4.4 (http://tree.bio.ed.ac.uk/software/figtree/).

Correlation analysis between GC content and genome size, as well as the ratio of core genome duplicated genes and genome size, was performed in R using the cor.test function with the Spearman's test. Group comparisons were assessed using the Kruskal–Wallis Rank Sum Test with the R function kruskal.test.

## Results

### Evolutionary history of the Fusarium species complexes

In this study, we incorporated a substantial number of reference species genomes obtained from the NCBI RefSeq database, encompassing all the *Fusarium* species complexes documented to date (Supplementary Table 1). Subsequently, we employed an extensive set of loci, which consisted of 559 conserved single-copy proteins, to establish a phylogenomic framework within the *Fusarium* genus for subsequent analyses.

The selection of these 559 proteins was based on three specific criteria: i) encoded by single-copy genes, ii) annotated as Nectriaceae proteins by the Eggnogmapper tool, iii) showing no duplicated KO annotation terms. These criteria were adopted to minimize the inclusion of paralogous proteins, which could potentially introduce noise into the phylogenetic reconstruction. The list of the selected proteins and their annotations can be found in (Supplementary Table 2).

**Fig. 1:**
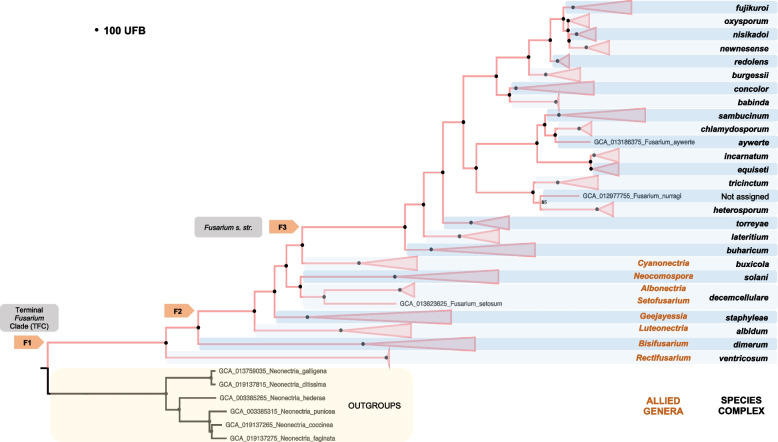
Maximum-likelihood phylogenomic tree based on 559 single-copy conserved proteins. The tree collapsed at the established *Fusarium* species complexes. The black circle (•) denotes 100% UFB support. The UFB value is indicated in the nodes with support below 100. *Neonectria* was included as an outgroup

Our reconstructed phylogenomic tree aligns with prior research, supporting *Fusarium* as a monophyletic group (100 UFB support). Furthermore, the tree illustrates all currently accepted species complexes as monophyletic groups with 100% UFB support (Fig. 1). The basal clades of the phylogenetic tree were *F. ventricosum* and *F. dimerum* species complexes, both branching between F1 anf F2 nodes. The two most basal clades of the F2 subdivision were *F. albidum* and *F. staphyleae* species complexes.

Subsequently, the tree delineates two major branches: one encompassing the *F. solani* and *F. decemcellulare* lineages and the other clade comprising the remaining species complexes. The most basal lineages within the latter branch are, in order, the *F. buxicola*, *F. buharicum*, *F. lateritium*, and *F. torreyae* species complexes. This final branch encompasses most of the described species complexes to date. Within this branch, we identify two primary lineages: one encompassing the species complexes *tricintum*, *heterosporum*, *incarnatum*, *equiseti*, *chlamydosporum*, and *sambucinum*, and the other including the species complexes *concolor*, *babinda*, *burgessi*, *redolens*, *newnesense*, *nisikadoi*, *oxysporum*, *claminii*, and *fujikuroi*. Supplementary Figure 1A, B, and C present the complete, uncollapsed phylogenomic tree comprising all *Fusarium* genomes analyzed.

### NCBI RefSeq database Fusarium genome quality analysis

We analyzed of various genome quality parameters within the database, including assembly length (genome size), assembly N50, largest scaffold, and scaffold count. Additionally, we performed a BUSCO genome quality control assessment, evaluating metrics such as completeness, single copy genes, duplicated genes, fragmented genes, and missing genes.

As depicted in the boxplots in (Supplementary Figure 2), genome statistics are only slightly dispersed for assembly length and BUSCO genome completeness. For the other assessed metrics, such as N50 and scaffold count, we observed a wider dispersion of values, indicating a heterogeneous performance in genome sequencing experiments. Notably, there was an approximately one-order-of-magnitude difference between the lower and upper limits of the boxplots, suggesting varying levels of success in the genome assembly process. Similar trends were observed in the BUSCO genome quality metrics, with some genomes performing poorly.

**Fig. 2:**
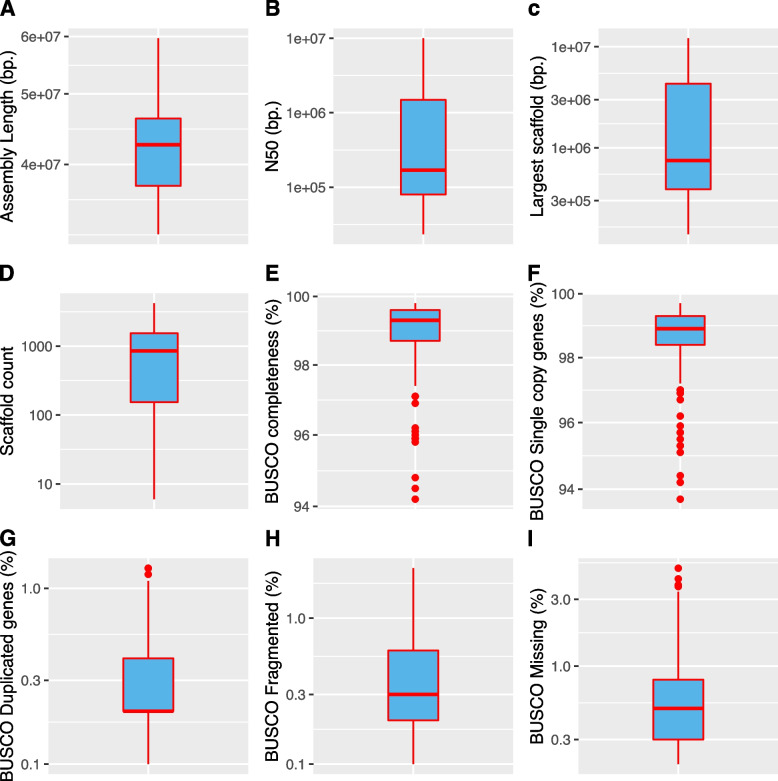
Box plot representation of the *Fusarium* reference genomes quality metrics after removing low-quality genomes. **A** Assembly length, **B** Assembly N50, **C** Largest scaffold length in bp., **D** Scaffold count, **E** BUSCO genome completeness, **F** BUSCO single copy genes detected, **G** BUSCO duplicated genes, **H** BUSCO fragmented genes and **I** BUSCO missing genes. Outliers are presented as black dots

To enhance the quality and reliability of subsequent analyses, we implemented genome quality thresholds: i) scaffold number (≤ 4,225) and ii) BUSCO metrics completeness and single copy genes (≥ 90%). Applying these filters led to the removal of 9 genomes from the dataset (For a detailed list of excluded genomes, please refer to Supplementary Methods). Assembly and BUSCO metrics were much less dispersed in the refined dataset comprising 216 *Fusarium* genomes, as illustrated in Fig. 2.

### Evolution of the Fusarium genome: GC ratio, size and core genome duplications

We categorized the RefSeq genomes based on their corresponding species complexes and generated box plots to depict variations in whole-genome GC content, GC content of the single-copy conserved genes CDS sequences, assembled genome size, and the fraction of duplicated core genes within each group. In the analysis of core-conserved CDSs GC content, we considered a subset of CDSs ranging between 4076 and 4480 per genome, representing approximately 50% of the expected CDS sequences in the *Fusarium* genome.

**Fig. 3:**
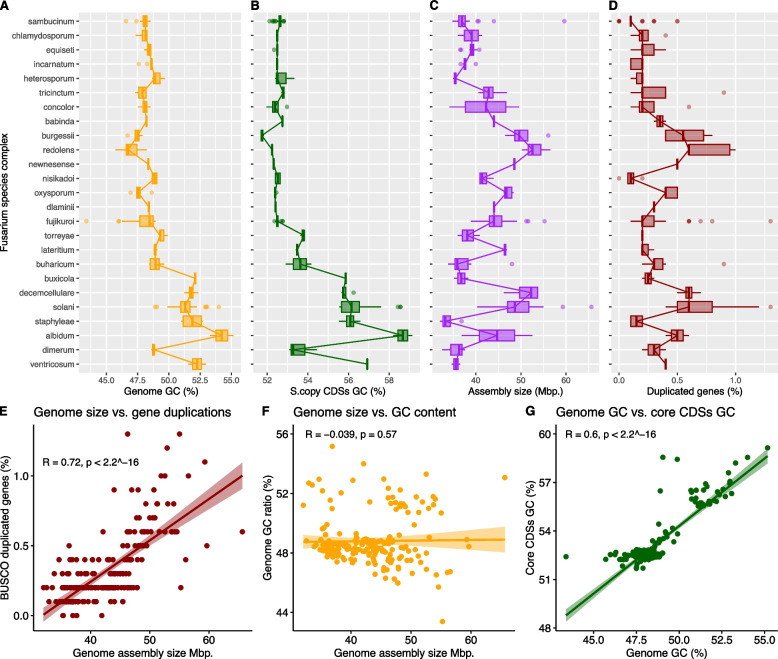
Evolution of the whole genome GC Content, core genes CDS sequences GC content, Genome Size, and Duplications of Core Genes in *Fusarium*. The species complexes are organized as presented in the collapsed tree, with the most ancestral clades positioned at the bottom of the graph. Lines connect the median values of the boxplots. Kruskal–Wallis rank sum test *p* < 2.2 × 10^-16. **A** Box plots illustrating the evolution of genome GC content. **B** Box plots demonstrate the evolution of core gene CDS sequences GC content, with analysis conducted on a subset of CDSs ranging between 4076 and 4480 per genome. **C** Box plots depicting the evolution of genome size. **D** Box plots displaying the evolution of the ratio of duplicated core genes. **E** Spearman correlation analysis that illustrates the relationship between genome size (x-axis) and the ratio of duplicated core genes (y-axis) in *Fusarium*. Each data point represents a species (*p*-value < 2.2 × 10^-16, rho = 0.720358). **F** Spearman correlation analysis that illustrates the relationship between genome size (x-axis) and GC content (y-axis) in *Fusarium* (*p*-value = 0.5707, rho = -0.03878989). **G** Spearman correlation analysis that illustrates the relationship between whole genome GC content (x-axis) and conserved core gene CDS sequences GC content (y-axis) in *Fusarium* (*p*-value < 2.2 × 10^-16, rho = 0.6038318)

To gain insights into the evolutionary trends of these genome features, we arranged the species complexes in the plots based on their positions in the phylogenomic tree (Fig. 3). The analysis revealed that variations in these genomic features depended on the studied group, and in most cases, genome disparities were narrower within each species complex compared to the entire genus. These findings were statistically significant for all three analyzed variables, with *p*-values below 2.2 × 10^-16 in all cases.

There are striking variations in GC content within the *Fusarium* genus, ranging from 43.4% to 55.2%. The basal clades to the F3 group generally exhibit higher GC content, exceeding 51% (species complexes *ventricosum*, *albidum*, *staphyleae*, *solani*, *decemcellulare*, and *buxicola*), except for the *F. dimerum* species complex. The remaining species complexes within the F3 group display median GC content values below 49.3% (Fig. 3A).

To elucidate the origins of differences in genome GC content (Fig. 3B), we conducted a comparative analysis of the median GC content within the coding sequences of core conserved genes in *Fusarium*, contrasting it with the overall genome GC content. For the purpose of this study, we defined core-genome CDSs as the single-copy CDSs identified by the BUSCO package, ranging between 4076 and 4480 CDSs per analyzed genome, constituting approximately 50% of the expected CDSs in the *Fusarium* genome.

Notably, the analysis revealed that while the median GC content of *Fusarium* genomes is 48.4%, the core conserved CDSs exhibit a higher median GC content of 52.5%. The GC content of the core conserved CDSs consistently remained above 51.5%.

This discrepancy highlights that coding sequences within the core conserved genes exhibit elevated GC content ratios compared to the median value of the whole genome, thereby contributing to higher GC ratios throughout the overall genome. This trend is prominently illustrated in Fig. 3A and B, where, across most species complexes, the GC content of the core gene CDSs aligns with the observed pattern in the whole genome data, albeit consistently displaying a higher median value. A Spearman correlation analysis further underscores this observation, demonstrating a robust correlation between the whole genome GC content and the GC content of core conserved CDSs (*R* = 0.6038318, *p* < 2.2 × 10^ − 16) (Fig. 3G).

Historically, the genus *Fusarium* has been described as having a broad range of genome sizes, ranging from 33 to 60 Mbp. However, our analysis reveals that genome sizes range from 32.05 to 65.63 Mb. Nevertheless, within each species complex, the variations are more discrete and show significant changes during evolution. The *F. redolens* species complex exhibits the largest genomes (median size 52.6 Mb), while the *F. staphyleae* complex shows the most reduced genome versions (median 33.25 Mb) (Fig. 3C).

As a complementary analysis, we investigated whether there is a relationship between genome size and duplication events in the *Fusarium* core genome. To do so, we quantified and plotted the proportion of duplicated core genes within each genome. As shown in Fig. 3D, duplication events of core genes vary depending on the species complex, with the *F. solani*, *F. decemcellulare*, and *F. redolens* complexes having the highest proportions at 0.6%. Additionally, the boxplot graph reveals a consistent trend between genome size and the proportion of core genes duplicated. To confirm this observation, we conducted a correlation analysis between genome size and duplicated genes. As seen in (Fig. 3)E, there is a strong correlation between genome size and the proportion of duplicated core genome genes (*R* = 0.72, *p* < 2.2 × 10^ − 16). In contrast, GC content does not exhibit such a correlation (*R* = 0.039, *p* = 0.57) (Fig. 3F).

### Genome conservation in Fusarium

**Fig. 4:**
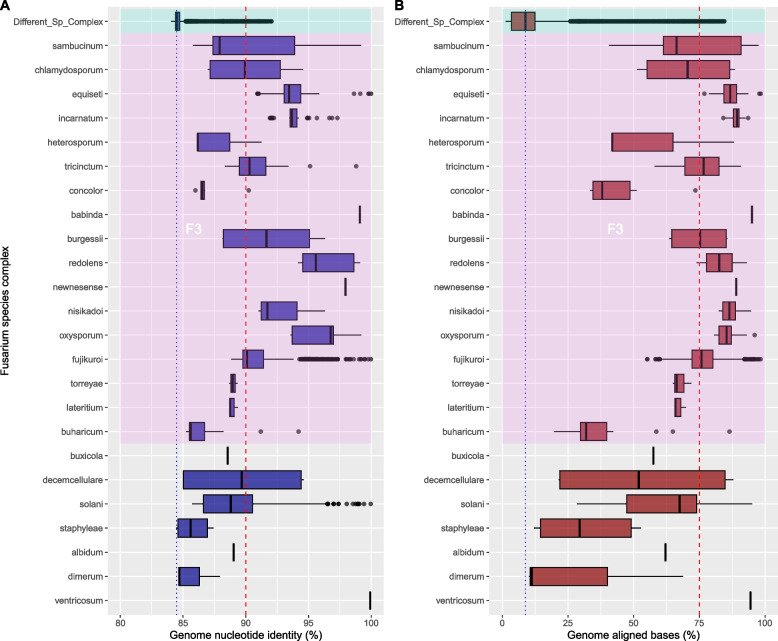
Genome Conservation Among *Fusarium* Species Complexes. **A** Box plots display variations in nucleotide identity across different genomes of the *Fusarium* species complexes. Comparisons between different species complexes are depicted in the ‘Different_Sp_Complex' category. The species complexes are organized as presented in the collapsed tree, with the most ancestral clades at the bottom of the graph. The dashed red line represents the median value when comparing within the same species complexes, while the dotted blue line signifies the median value for comparisons between species complexes. Kruskal–Wallis rank sum test *p* < 2.2 × 10^-16. The pink rectangle the depicts the species complexes of the *Fusarium* senso stricto clade (F3). **B** Box plots display variations in genome-aligned bases across different genomes of the *Fusarium* species complexes. Comparisons between different species complexes are depicted in the ‘Different_Sp_Complex' category. The species complexes are organized as presented in the collapsed tree, with the most ancestral clades at the bottom of the graph. The dashed red line represents the median value when comparing within the same species complexes, while the dotted blue line signifies the median value for comparisons between species complexes. Kruskal–Wallis rank sum test *p* < 2.2 × 10^-16. The pink rectangle the depicts the species complexes of the *Fusarium* senso stricto clade (F3)

**Fig. 5:**
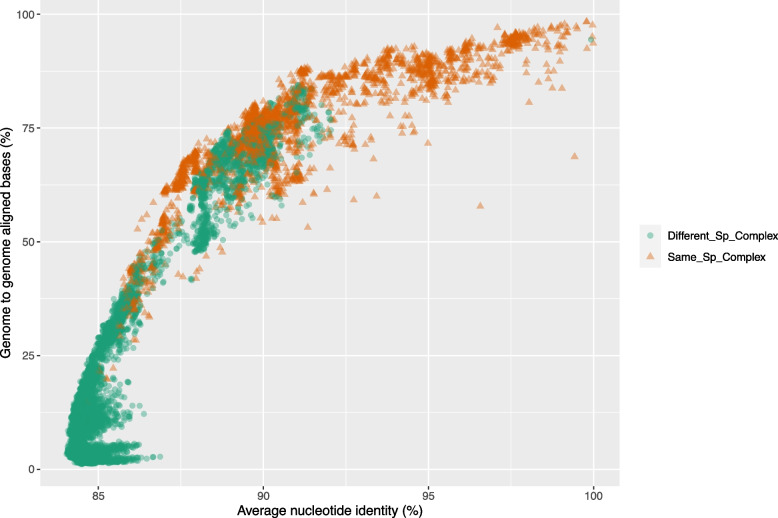
Scatter Plot Analysis of the Relationship Between Nucleotide Identity and Genome-to-Genome Aligned Bases in *Fusarium*. This scatter plot illustrates the correlation between nucleotide identity and the proportion of genome-to-genome aligned bases across different genomes of the *Fusarium* reference species. Each data point represents a pairwise comparison between two genomes, with nucleotide identity on the x-axis and the proportion of aligned genome bases on the y-axis. The color and shape code distinguishes comparisons within the same species complexes (intra-species-complex, shown in orange triangles, Same_Sp_Complex) and comparisons between species complexes (inter-species-complex, shown in green circles, Different_Sp_Complex). Kruskal–Wallis rank sum test *p* < 2.2 × 10^-16

To gain a deeper understanding of genome conservation among *Fusarium* reference species, we employed a genome-to-genome alignment strategy using the MUMMER program. Our analysis encompassed the calculation of both the proportion of the aligned genomic blocks and the nucleotide identity within them. Median values of the proportion of genome-aligned bases within each group exhibited a range from 11.23% to 94.99%, with a decrease to 8.82% when comparing between species complexes. In contrast, the median values of nucleotide identity within each complex spanned from 84.71% to 99.92%, with a median of 84.50% for comparisons between species complexes (Fig. 4). The overall median values for genome-aligned bases and nucleotide identity, when comparing genomes within each corresponding species complex, were 75% and 90%, respectively. These differences in the fraction of genome-aligned bases and the average nucleotide identity were statistically significant, with a *p*-value < 2.2 × 10^-16. In addition, we conducted a scatter plot analysis comparing the proportion of aligned genome versus nucleotide identity, both within and between species complexes. This analysis revealed a general trend where inter-species-complexes exhibited lower proportions of genome-aligned blocks, while intra-species-complexes comparisons tended to have a larger proportion (Fig. 5).

**Fig. 6:**
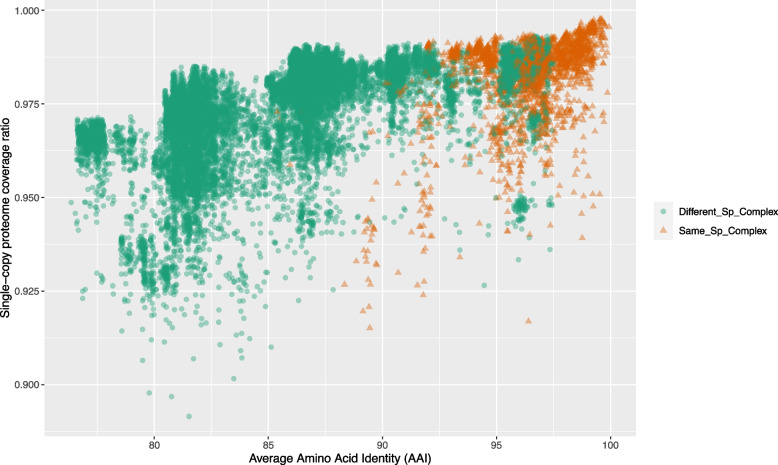
Scatter Plot Analysis of the Relationship Between Average Amino Acid Identity (AAI) and Core Proteome Coverage in *Fusarium*. This scatter plot illustrates the correlation between Average Amino Acid Identity (AAI) and the coverage of the core proteome across different proteomes of the *Fusarium* reference species. Each data point depicts a pairwise comparison between two proteomes, with the AAI score on the x-axis and the ratio of the proteome coverage on the y-axis. Data points are color-coded and marked with different shapes to distinguish comparisons within the same species complexes (intra-species-complex, shown in orange triangles, Same_Sp_Complex) and comparisons between species complexes (inter-species-complex, shown in green circles, ‘Different_Sp_Complex)

In addition, we sought to quantify the extent of conservation within the core proteome of the *Fusarium* genus. To achieve this, we annotated and compared all the conserved single-copy proteins using the BUSCO software. The average amino acid identity (AAI) of these conserved single-copy proteins for each genome and the overall coverage ratio of the single-copy proteome are visualized in the scatter plot presented in Fig. 6. The analysis demonstrates that the single-copy core proteome of *Fusarium* is markedly well-conserved among the examined reference genomes, with at least 90% of it present in nearly all tested species and displaying an AAI value exceeding 70%. Notably, when comparing within or between species complexes, intra-lineage comparisons yielded higher AAI values, consistently above 88%, for nearly all proteomes, and coverage ratios exceeding 0.91.

**Fig. 7:**
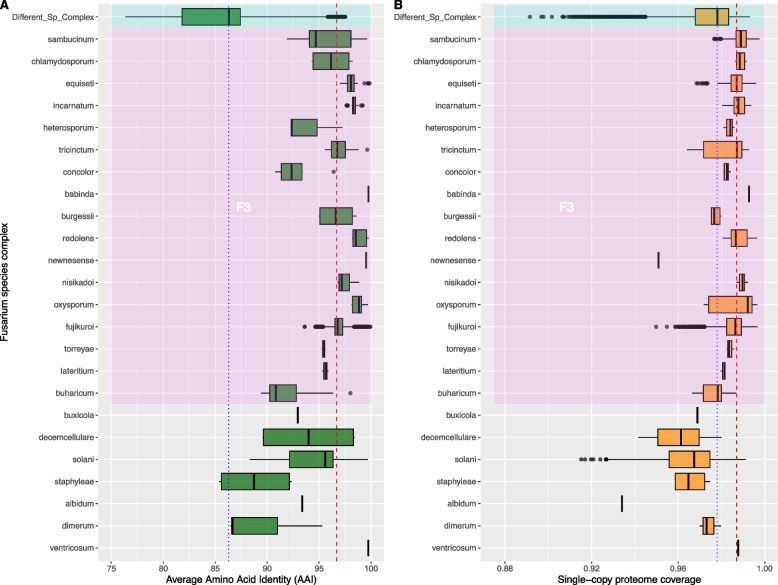
Core proteome Conservation Among *Fusarium* Species Complexes. **A** Box plots display variations in Average Amino Acid Identity (AAI) across different genomes of the *Fusarium* species complexes. AAI comparisons between different species complexes are depicted in the ‘Different_Sp_Complex' category. The species complexes are organized as presented in the collapsed tree, with the most ancestral clades at the bottom of the graph. The dashed red line represents the median value when comparing within the same species complexes, while the dotted blue line signifies the median value for comparisons between species complexes. Kruskal–Wallis rank sum test *p* < 2.2 × 10^-16. **B** Box plots display variations in the coverage of the core proteome across different genomes of the *Fusarium* species complexes. AAI comparisons between different species complexes are depicted in the ‘Different_Sp_Complex' category. The species complexes are organized as presented in the collapsed tree, with the most ancestral clades at the bottom of the graph. The dashed red line represents the median value when comparing within the same species complexes, while the dotted blue line signifies the median value for comparisons between species complexes. Kruskal–Wallis rank sum test *p* < 2.2 × 10^-16. The pink rectangle the depicts the species complexes of the *Fusarium* senso stricto clade (F3) in both panels

A more detailed analysis, utilizing boxplots and differentiating the F3 lineage within the *Fusarium* genus, reveals that the basal species complexes in the F3 lineage tend to exhibit lower AAI and proteome coverage ratios compared to the F3 lineage as a whole. Specifically, the median AAI value for inter-complex comparisons was 86.32%, whereas intra-complex comparisons yielded a median AAI value of 96.32%. Interestingly, the core proteome coverage was similar in both comparison groups, with values of 0.98 and 0.99 for inter-species complexes and intra-species complexes comparisons, respectively (Fig. 7). These differences in the AAI score and core proteome coverage ratio were statistically significant, obtaining a. *p*-value < 2.2 × 10^-16.

**Fig. 8:**
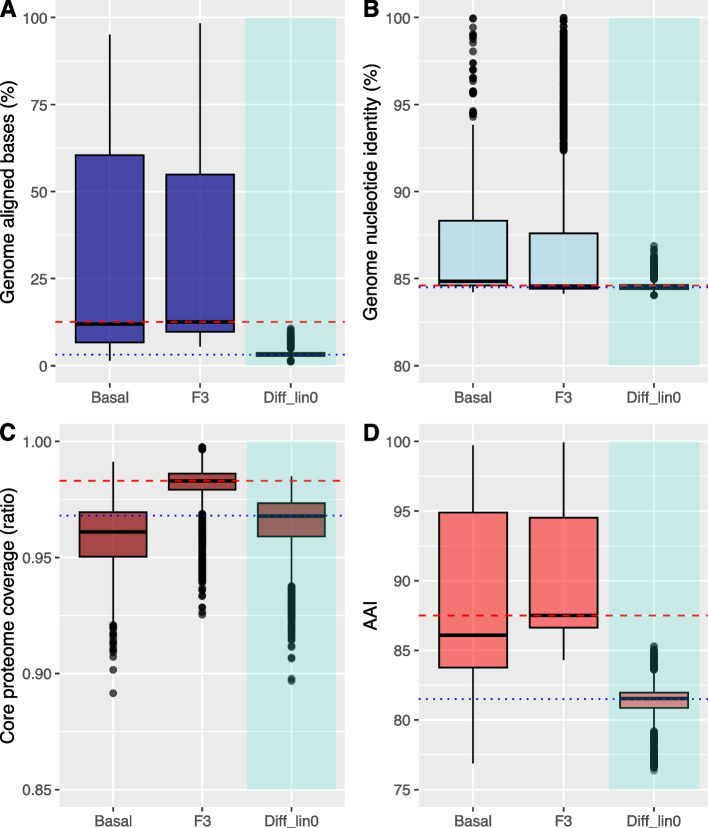
Genome and Core Proteome Conservation Among *Fusarium* Basal (Allied genera and the F3 (*Fusarium* sensu stricto) Species. Box plots display variations in genome-aligned bases (**A**) and nucleotide identity (**B**) across different genomes of the *Fusarium* basal reference species (‘allied’ genera—Basal), the *Fusarium* sensu stricto—F3 reference species (F3), and between the basal and F3 reference species (‘Different_Sp_Complex). The dashed red line represents the median value when comparing within the F3 species, while the dotted blue line signifies the median value for comparisons between F3 and Basal reference species. Kruskal–Wallis rank sum test *p* < 2.2 × 10^-16

To provide a more comprehensive understanding of genome conservation within the *Fusarium* genus, especially in relation to the F3 hypothesis, we categorized the genomes into two primary groups: the F3 lineage and the basal taxa. We then conducted genome-to-genome alignment comparisons to assess their conservation patterns. As depicted in Fig. 8A, genomes within the F3 hypothesis clade demonstrated a notably higher level of conservation, with a median alignment success rate of 12.6%. In contrast, when comparing alignment coverage between the F3 lineage and the basal taxa, there was a significant decline to 3.19%. Within the basal taxa, the median proportion of the aligned genome reached 12%. Interestingly, the average nucleotide identity of the aligned genome blocks displayed similar values across all three tested groups, measuring at 84.8% for basal taxa, 84.6% for the F3 lineage, and 84.5% for inter-lineage comparisons (see Fig. 8B).

Furthermore, when examining the conservation of the core proteome, subtle yet significant differences emerged. The AAI value within the F3 group reached 87.5, whereas it dropped to 81.5 for comparisons between the F3 and basal genera species. In terms of core proteome coverage, the median value within the F3 group was 0.983, while it reduced to 0.968 when comparing the F3 group with basal genera species. Importantly, all comparisons among the three groups yielded statistically significant results, with *p*-values consistently below *p* < 2.2 × 10^-16 in all cases.

## Discussion

Taxonomy based on morphological or phenotypical characteristics of microscopic organisms has been exceptionally challenging since their discovery more than three centuries ago [9, 34]. Although classical taxonomic methods prompted progress for *Fusarium* studies, major concerns emerged with the advent of molecular systematics [20]. Thankfully, the recent advances in Next-generation sequencing (NGS) and bioinformatics allowed mycologists to start clarifying the complex relationships within this fungal taxon and to reveal novel species [2, 12, 21, 35]. In the case of *Fusarium*, advances are remarkable, leading to the identification of approximately 400 species and a better understanding of the genus's evolutionary history [8]. As a first step in this work, we assessed the quality of the genomes available in the NCBI RefSeq database for *Fusarium* species. While most of these genomes are reliable, some exhibited poor quality indicators. These low-quality genomes should be avoided as references for comparative genomic analysis, emphasizing the need for new, trustworthy genome projects for these species.

Our phylogenetic analysis, based on 559 conserved single-copy proteins, confirms that the currently recognized 28 species complexes of the *Fusarium* genus form well-supported monophyletic groups. Furthermore, 198 (90%) nodes in our tree have a support level of ≥ 95%, with nodes F1, F2, and F3 achieving 100% support [35].

The depicted phylogeny also aligns with more recent studies, supporting the concept of the 'broad' *Fusarium* clade, which encompasses 17 species complexes and 11 allied genera [36]. The tree topology also supports the 'narrow' concept of the genus *Fusarium* sensu stricto, which includes only the 17 species complexes, often referred to as the F3 hypothesis [19, 36–38]. The branch lengths observed in the phylogenomic tree further indicate a wider evolutionary divergence between the 'allied' genera and the species at the F3 node.

Concordance factors (gcf and scf) were calculated to refine the accuracy of our conclusions regarding phylogenetic reconstructions. For instance, F1 (gcf 98.8%, scf 91.7%), F2 (gcf 55%, scf 46.9%), and F3 (gcf 83.4%, scf 71.6%) showed significant support. A gcf value above 50% suggests that more than half of the ortholog proteins used support the node positions. It's worth noting that even low support values do not necessarily indicate an indeterminate phylogeny; instead, they provide information about the degree of relationship or congruence, helping elucidate the evolutionary history of the species [39].

We have identified topological differences when comparing our phylogenomic results to previously published phylogenies, particularly at the basal nodes. One significant difference involves the positioning of the *F. ventricosum* species complex. In our phylogenetic analyses, the *F. ventricosum* complex emerges as the most ancestral taxon within *Fusarium*, which contrasts with the hypothesis presented by Geiser and O'Donnell [2, 12, 35, 40]. In those phylogenies, the *F. ventricosum* and *F. dimerum* species complexes were grouped as an ancestral monophyletic clade, albeit with low support, such as 64% ML-BS, less than 50 maximum parsimony bootstrap (MP-BS), and Bayesian posterior probability (B-PP) of 1.0 [35]. O'Donnell in 2013 reported ML-BS and BP-BS values below 70%, and Geyser (2021) noted BS values under 70 and BPP below 0.99, with a gene concordance factor (gcf) of 0, indicating minimal support for this node in their analyses. In all these cases, the authors acknowledged the necessity for additional studies to clarify the position of these complexes, given the low support values. Conversely, in other phylogenies presented by Lombard et al. [37], Gräfenhan et al. [19], Han et al. [36], and Chen Y.P et al. [41], the *F. ventricosum* complex is placed as ancestral to the *F. dimerum* complex, in concordance to our findings.

Regarding the basal clades of the genus, F1 hypothesis, we observed four basal complexes, each with strong support: i) ventricosum complex (allied genus *Rectifusarium*) node is well-supported with UFB 100%, gcf 98.8%, and scf 91.7%, ii) *F. dimerum* complex (allied genus *Bisifusarium*) node shows support with UFB 100%, gcf 32.9%, and scf 38.4%, iii) *F. albidum* complex (allied genus *Luteonectria*) node has support with UFB 100%, gcf 55%, and scf 46.9%, and iv) *F. staphyleae* complex (allied genus *Geejayessia*) node receives BS 100%, gcf 26.4%, and scf 35.9%. Within this node, we find the allied genus *Nothofusarium* forming a monophyletic clade with *Geejayessia*. This genus is found in the phylogenies presented by Han et al. [36] and Chen et al. [41] with robust bootstrap support.

The position of the *F. staphyleae* species complex has been debated. O'Donnell et al. [12] proposed it as the 4th most ancestral clade, but its position lacked support, warranting further analysis. In contrast, Geyser et al. 2021 [40] positioned the *F. staphyleae* complex within a clade containing the *F. solani*, *F. decemcellulare*, and *F. buxicola* species complexes. However, this placement also had relatively low support (81% ML-BS and 0.99 BPP). Our phylogenomic tree supports the hypothesis that *Setofusarium setosum* (allied genus *Setofusarium*) and *F. staphyleae* (allied genus *Albonectria*) represent ancestral lineages of the *F. solani* (allied genus *Neocosmopora*) and *F. buxicola* (allied genus *Cyanonectria*) species complexes.

In summary, our results support the hypothesis that the most ancestral clades within the *Fusarium* genus, in sequence, are *F. ventricosum*, *F. dimerum*, *F. albidum*, and *F. staphyleae* species complexes. They are followed by the *F. solani* + *decemcellulare* clade and then the *F. buxicola*, *F. buharicum*, *F. lateritium*, and *F. torreyae* complexes. Notably, these last four complexes share the same topology and receive 100% UFB support in both our phylogeny and the one proposed by Geiser et al. in 2021 [40]. Subsequently, lineages beyond *F. buxicola* (allied genus *Cyanonectria*) form the F3 clade, *Fusarium* s. str., comprising 17 complexes of *Fusarium* species. This taxonomic proposal aligns with the phylogenies presented by Han et al. [36] and Chen et al. [41].

Regarding the species complexes *F. sambucinum* (FSAMSC), *F. incarnatum*-*equiseti* (FIESC), *F. babinda* (FBSC), *F. concolor* (FCOSC), *F. burgessi* (FBurSC), and *F. redolens* (FRSC), no topological differences are observed concerning the phylogenies presented by Geiser (2021) [40], Chen (2023) [41], and S. L. Han (2023) [36]. These nodes in our analysis received strong support with 100% UFB. While Geyser et al. reported poor support for the *F. concolor* clade, our results provide robust evidence for the monophyly of this species complex, with 100% UFB support.

We also found that the *F. newnesense* (FnewSC), *F. nisikadoi* (FNSC), *F. oxysporum* (FOSC), and *F. fujikuroi* (FFSC) species complexes form a monophyletic clade in agreement with Geiser et al. (2021) [40], Han et al. [36], Chen et al. [41], and Crous et al. [38]. However, our phylogeny suggests a different evolutionary history within this clade. In our analysis, *F. fujikuroi* emerges as the most ancestral lineage, followed by *F. oxysporum* and the *F. nisikadoi* + *newnesense* branches. Geiser et al. (2021) reported less consistent support for this clade, with some bootstrap values below 90% [40].

The evolutionary analysis of genome GC content suggests that ancestral *Fusarium* lineages, the ‘allied’ genera, have higher GC content ratios, exceeding 50%, while most modern clades in the F3 clade have shown a reductive trend in this index, with values dropping to 47–48% in most species complexes.

In delving into the origin of GC variations within *Fusarium* genomes, we sought to discern whether these variations predominantly arise from accessory genome elements rather than representing a global evolution trend toward lower GC ratios. To address this, we specifically examined the GC content evolution of the conserved core CDS sequences. Our results vividly illustrate that the GC content evolution in *Fusarium* genomes aligns with a similar trend observed between the whole genome and the core gene CDS sequences. A statistically significant positive correlation analysis further substantiates this alignment.

These findings imply that while unstable repetitive genome elements may exert some influence on overall genome GC ratios, the conserved core genome exhibits a parallel trend toward an accumulation of more AT bases. This aligns with the overarching pattern observed across the entire genome.

While ancestral lineages, such as *F. ventricosum* and *F. dimerum*, showed smaller genomes below 38 Mb, the remaining species complexes underwent significant gains and losses in genomic content during evolution, resulting in genome sizes fluctuating between 32 and 66 Mbp. Notably, substantial genome gains occurred in basal taxa and within the F3 clade, particularly in the *F. albidum*, *F. solani*, *F. decemcellulare*, *F. oxysporum*, *F. newnesense*, *F. redolens*, and *F. burgessii* complexes. This phenomenon has been previously discussed in other studies where the gain and loss of accessory chromosomes drove changes in genome sizes [42]. However, our analysis revealed that these genome gains strongly correlate with duplications of conserved genes within the core genome. A similar phenomenon of genome expansion associated with duplications of ancestral genes has been previously observed in Archaea [43].

As a result, genome size and GC content appear to be distinct characteristics within each species complex.

Genome conservation in *Fusarium*, as measured by the proportion of the genome aligned between different species complexes, appears to be relatively low, with only approximately 12% of the genome aligning within the species complexes of the basal clade (‘allied’ genera) or within the species complexes of the F3 clade (*Fusarium* sensu stricto). However, when comparing these two clades, the alignment proportion drops to nearly a quarter, indicating a more distant evolutionary relationship between these two groups.

Conversely, when examining the core proteome of the genus, a notably higher level of conservation becomes evident, with at least 90% of the proteins detected in almost all tested reference strains. Furthermore, the average amino acid identity score (AAI) exceeds 75%, with a median value of 86% when making comparisons between species complexes. One plausible interpretation of this phenomenon aligns with previous reports, suggesting that the basal groups of the F3 clade exhibit a significant evolutionary distance from the *Fusarium* sensu stricto group (F3). This provides support for arguments favoring the classification of the basal groups into different genera outside the genus *Fusarium* [36, 38, 44].

An intriguing observation arises from our analysis of genome and proteome conservation, depicted in the scatter plots in Figs. 5 and 6. Several species exhibit analogous conservation profiles for their genomes and proteomes, highlighting that, regardless of whether they belong to the same or different species complexes. This discovery suggests that certain species, traditionally grouped within the same species complex, exhibit evolutionary distances and molecular divergences comparable to those observed between species categorized to different complexes. From a genomic standpoint, this raises questions about potential challenges within the ongoing classification framework of the *Fusarium* genus.

## Conclusions

Our study represents a significant step in understanding the taxonomy, evolution, and genome dynamics within the *Fusarium* genus. The advent of molecular systematics, coupled with recent advances in NGS and bioinformatics, has provided us with invaluable tools to tackle the intricate relationships within this fungal taxon. Furthermore, our results indicate a broader evolutionary divergence between the 'allied' genera (basal clades) and the species within the F3 clade.

Our evaluation of the quality of genomes in the NCBI RefSeq database for *Fusarium* species highlights the importance of reliable reference genomes for comparative genomic analyses. While most genomes are dependable, some exhibit poor quality indicators, emphasizing the need for trustworthy genome projects for these species.

Our findings also shed light on the evolution of genome GC content and genome size within *Fusarium* species complexes. Ancestral lineages and some modern clades exhibit distinct patterns in these genomic characteristics. Notably, genome expansions correlate strongly with duplications of ancestral conserved genes within the core genome.

While genome conservation within *Fusarium* species complexes appears relatively low at the genomic level, the core proteome exhibits a notably higher level of conservation.

### Supplementary Information

**Supplementary Material 1. **

**Supplementary Material 2. **

**Supplementary Material 3.**

**Supplementary Material 4.**

**Supplementary Material 5.**

**Supplementary Material 6.**

## Data Availability

All the data used in this study is publicly available in the NCBI DATASETS database (https://www.ncbi.nlm.nih.gov/datasets/taxonomy/5506/). The detailed list of accessions is described in the methods section.
