# Supplementary material for: An evolutionary view of the Fusarium core genome
Source: BMC Genomics. 2024 Mar 22;25:304. doi: 10.1186/s12864-024-10200-w (PMC10958916; doi:10.1186/s12864-024-10200-w)
Supplement: Supplementary file 1 — Supplementary Material 1. [file 12864_2024_10200_MOESM1_ESM.docx]

**Supplementary methods.**

Genomes excluded from the initial dataset due to quality issues (n=9):

GCA_001680505-*Fusarium*_cuneirostrum

GCA_001680625-*Fusarium*_azukicola

GCA_001680685-*Fusarium*_brasiliense

GCA_011036685-*Fusarium*_cf_nygamai

GCA_012978535-*Fusarium*_sp_NRRL_62957

GCA_013186395-*Fusarium*_sp_NRRL_62944

GCA_0131864252-*Fusarium*_sp_NRRL_62610

GCA_0134167852-*Fusarium*_sp_BWC1

GCA_017656815_Fusarium_meridionale_x_Fusarium_asiaticum

**Supplementary Figure 1.** Maximum-likelihood phylogenomic tree based on 559 single-copy conserved proteins, presented as an uncollapsed tree divided into three subfigures, 1A, 1B, and 1C. The black circle (•) denotes 100% UFB support. Nodes with support below 100 display the UFB value. *Neonectria* was included as an outgroup.

**Supplementary Figure 2.** Box plot analysis of the *Fusarium* reference genome quality metrics of all 225 genome assemblies downloaded from the NCBI datasets database. The analyzed metrics include **A.** Assembly length, **B.** Assembly N50, **C.** Largest scaffold length in bp., **D.** Scaffold count, **E.** BUSCO genome completeness, **F.** BUSCO single copy genes detected, **G.** BUSCO duplicated genes, **H.** BUSCO fragmented genes, and **I.** BUSCO missing genes. Outliers are presented as black dots.

**R Scatter plots method.**

To generate the scatter plots of the AAI and genome-to-genome alignments we used GGPLOT2 in R studio (v.4.1.3) using the following commands:

#AAI

FusariumAAI <- read.csv("PAPER3_EDIT3_TABLA_w_LINAJES_AAI_Fusarium_genus_Filter3.csv", header = TRUE, sep = ",", dec = ".")

ggplot(FusariumAAI, aes(x=AAI, y=Proteome_cov, color=LINEAGE2, shape=LINEAGE2)) + geom_point(size=2, alpha=0.4) +

scale_color_brewer(palette="Dark2") + ylab(" Single-copy proteome coverage ratio ") + xlab(" Average Amino Acid Identity (AAI) ") + theme( legend.title = element_blank() )

## DNADIFF genome-to-genome alignments

DNADIFFdataCNSG <- read.csv("PAPER3_EDIT2_FILTER3_TABLAfullDNADIFF_SAMElineage_Fusarium.csv", header=TRUE, sep = ",", dec = ".")

ggplot(DNADIFFdataCNSG, aes(x=AvgIdentity, y=AlignedBases, color=LINEAGE2, shape=LINEAGE2)) + geom_point(size=2, alpha=0.4) +

scale_color_brewer(palette="Dark2") + xlab(" Average nucleotide identity (%) ") + ylab(" Genome to genome aligned bases (%) ") + theme( legend.title = element_blank())
