# Supplementary figures and images for: An evolutionary view of the Fusarium core genome
Source: BMC Genomics. 2024 Mar 22;25:304. doi: 10.1186/s12864-024-10200-w (PMC10958916; doi:10.1186/s12864-024-10200-w)

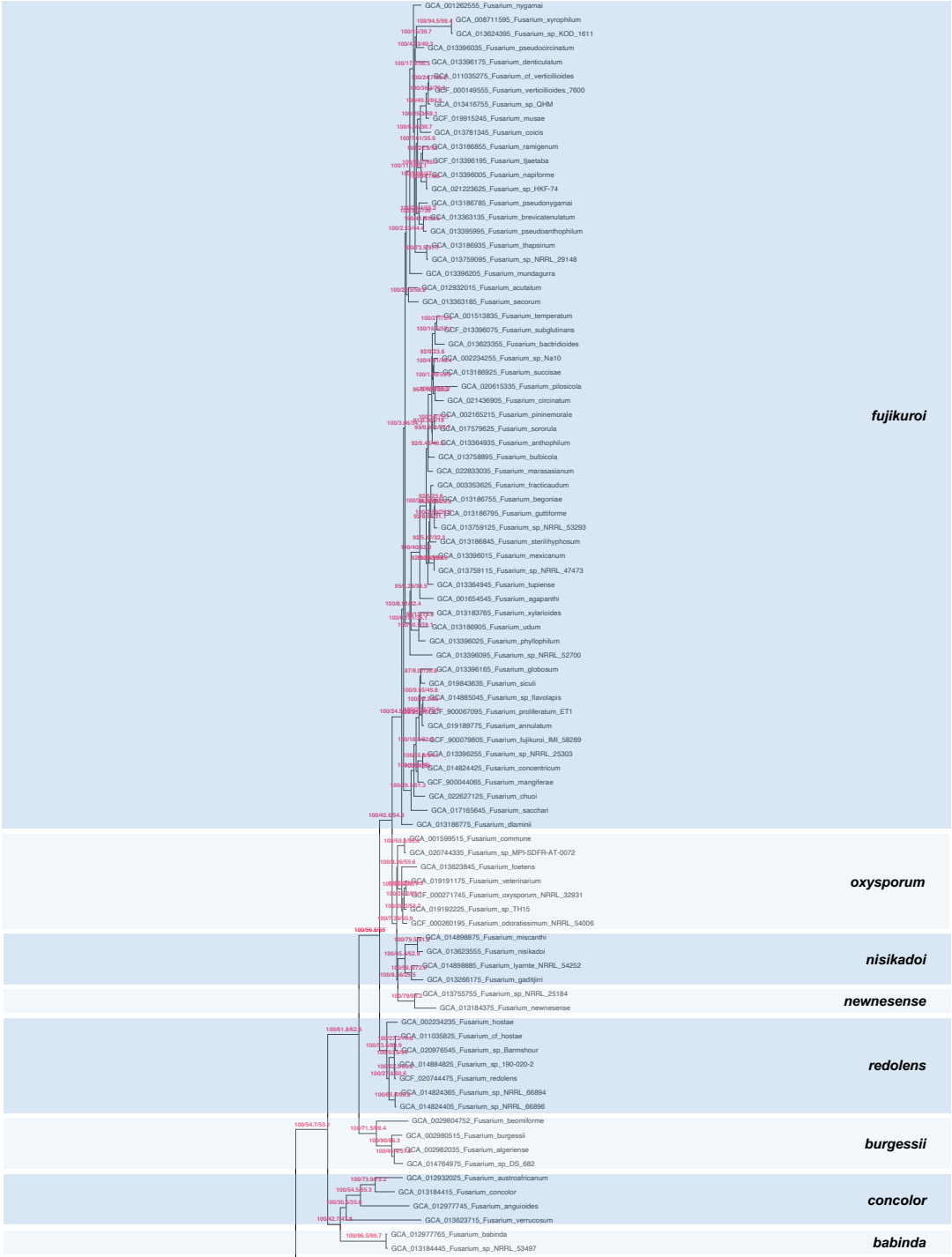

**Fusarium s. str.**

Supplement: Supplementary file 2 — Supplementary Material 2. [file 12864_2024_10200_MOESM2_ESM.zip › Supplementary_Figure_1A.pdf]

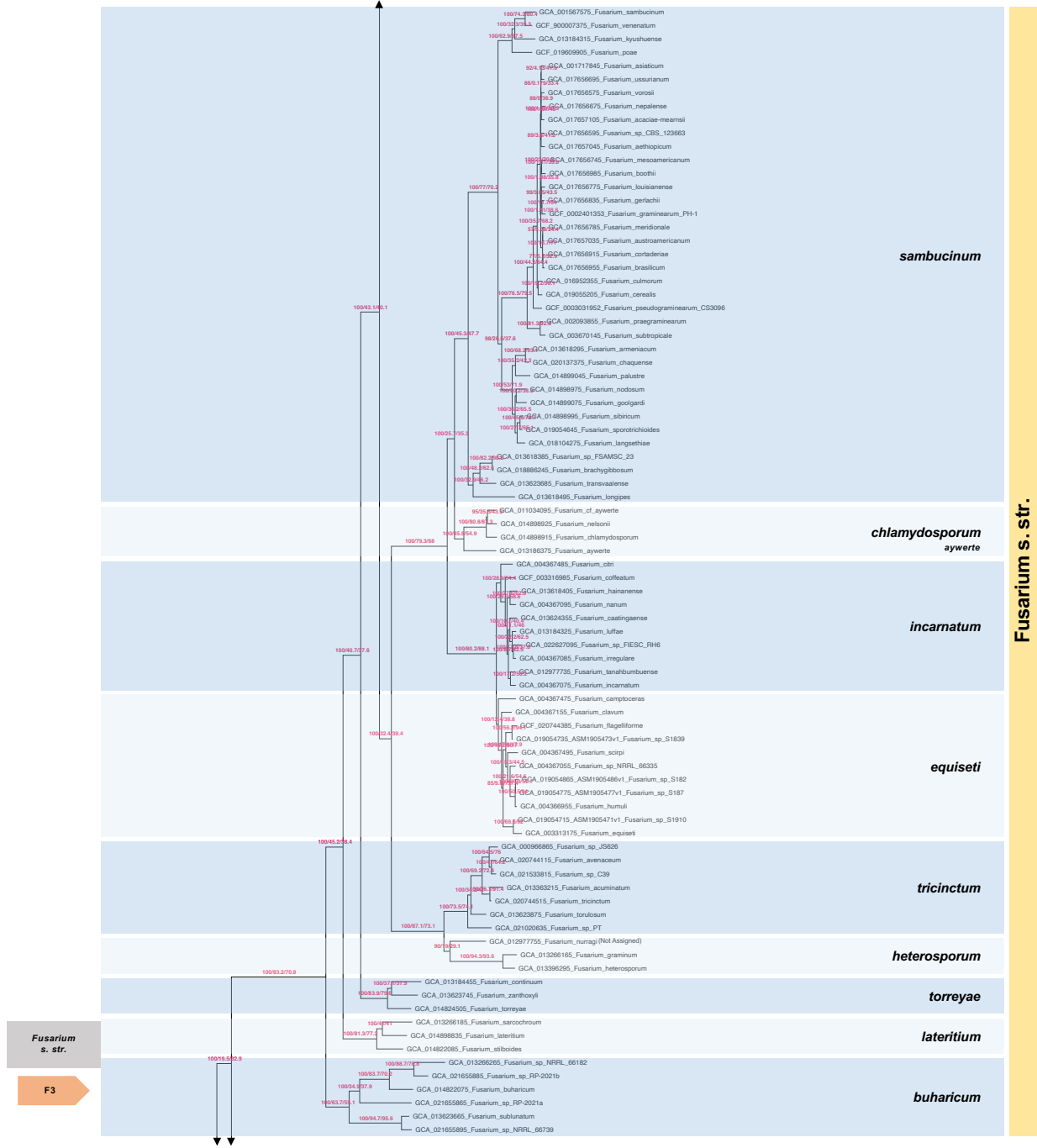

Supplement: Supplementary file 2 — Supplementary Material 2. [file 12864_2024_10200_MOESM2_ESM.zip › Supplementary_Figure_1B.pdf]

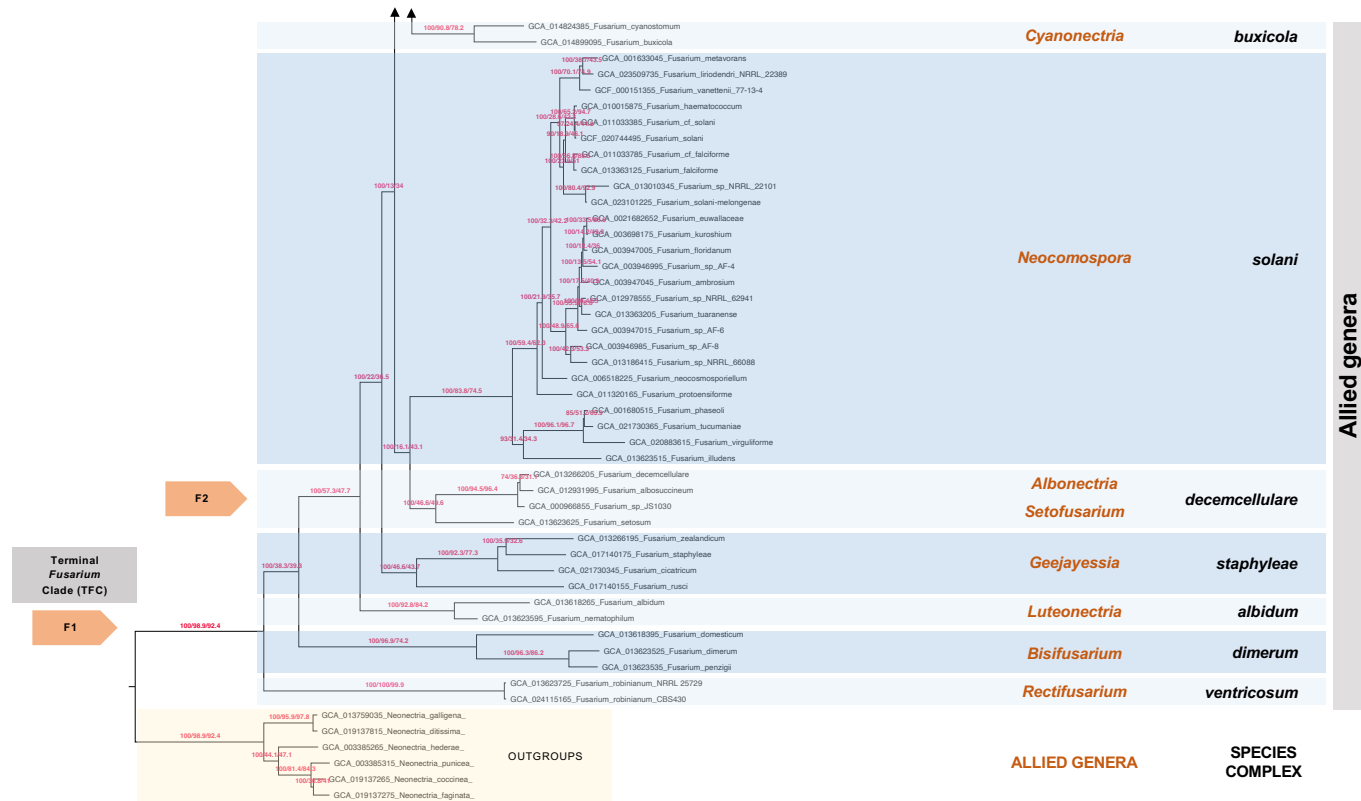

Supplement: Supplementary file 2 — Supplementary Material 2. [file 12864_2024_10200_MOESM2_ESM.zip › Supplementary_Figure_1C.pdf]

**A**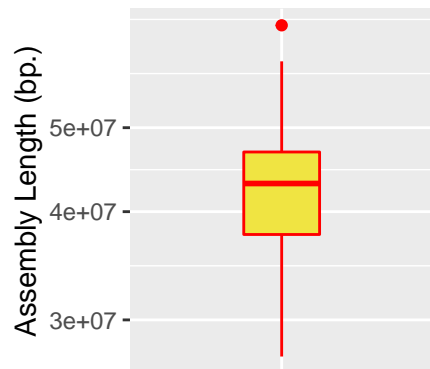**B**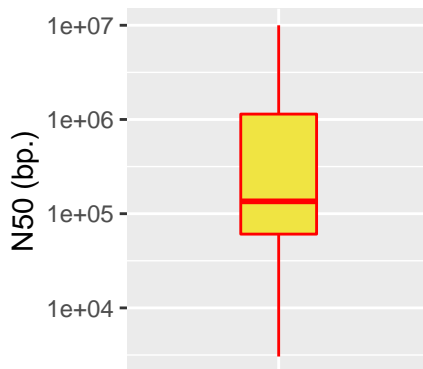**C**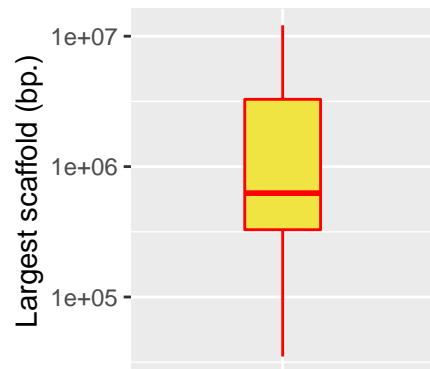**D**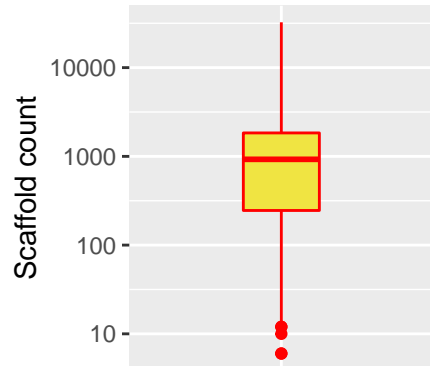**E**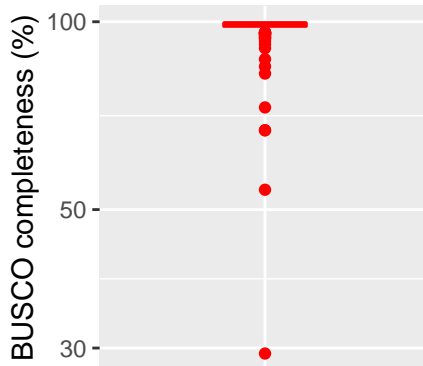**F**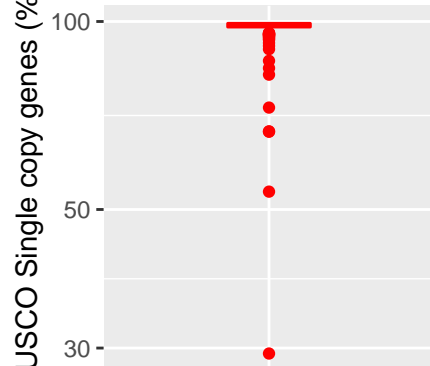**G**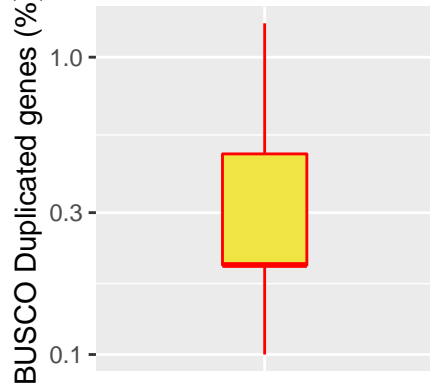**H**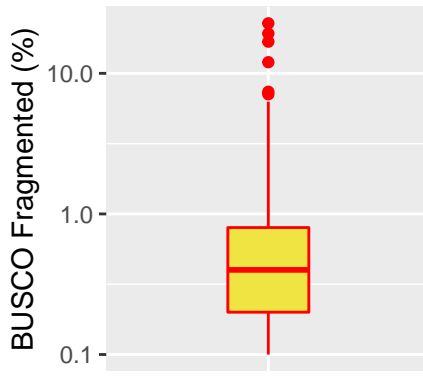**I**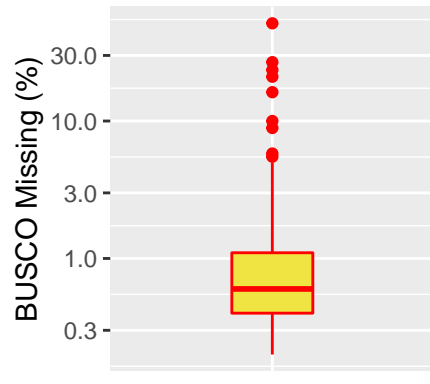

Supplement: Supplementary file 3 — Supplementary Material 3. [file 12864_2024_10200_MOESM3_ESM.pdf]
